# Medication Adherence and the Role of Pictograms in Medication Counselling of Chronic Patients: a Review

**DOI:** 10.3389/fphar.2021.582200

**Published:** 2021-08-19

**Authors:** Piotr Merks, Jameason Cameron, Krzysztof Bilmin, Damian Świeczkowski, Tomira Chmielewska-Ignatowicz, Tomasz Harężlak, Katarzyna Białoszewska, Katarina Fehir Sola, Miłosz J Jaguszewski, Regis Vaillancourt

**Affiliations:** ^1^ Faculty of Medicine, Collegium Medicum, Cardinal Stefan Wyszyński University, Warsaw, Poland; ^2^ Department of Pharmaceutical Technology, Collegium Medicum, Nicolaus Copernicus University, Toruń, Poland; ^3^ Department of Pharmacy, Children’s Hospital of Eastern Ontario, Centre Hospitalier pour Enfants de L’est de L'Ontario, Ottawa, ON, Canada; ^4^ First Department of Cardiology, Medical University of Gdansk, Gdańsk, Poland; ^5^ Department of Paediatric Dentistry, Medical University of Warsaw, Warsaw, Poland; ^6^ Pharmacy of Bjelovar, Bjelovar, Croatia

**Keywords:** pictograms, medication adherence, pharmaceutical care, medication counselling, health literacy

## Abstract

Pharmaceutical care requires a patient-centered approach, focusing on the ability of patients to understand drug-related information and follow the instructions delivered by pharmacists as well as other health-care providers included in the circle of care. With the goal of ensuring the prescribed use of medications, called medication adherence, health-care providers have to consider many risk factors such as geography (culture), social economic status, age, and low literacy that may predispose patients to non-adherence, and considerations have to be made for chronic patients living with life-long disease states. The aim of this review is to provide a balanced and comprehensive review outlining a number of different medication counselling and education approaches that have been used to try to improve medication adherence and health outcomes with the use of clear and concise graphic illustrations—called pictograms. By highlighting the current landscape of the general use and efficacy of pharmaceutical pictograms to aid in the knowledge and recall of drug-related information, as well as outlining specific medication adherence outcomes with pharmaceutical pictograms in chronic patients, the current review describes the need for health-care providers to move beyond the traditional didactic methods of oral and verbal communication with patients regarding medication-taking behavior.

## Introduction

Pharmaceutical care requires a patient-centered approach, focusing on the ability of patients to understand drug-related information and follow instructions delivered by pharmacists as well as other health-care providers included in the circle of care. Pharmaceutical care was defined by Hepler and Strand as the responsible provision of drug therapy for the purpose of achieving specific drug-related outcomes (i.e., cure a disease, eliminate or reduce symptomatology, arrest or slow disease progression, or prevent disease onset) as well as improve overall wellbeing and quality of life of the patient (Hepler and Strand, 1990). Pharmaceutical care involves a multidirectional communication process whereby the pharmacist cooperates with the patient, physician, and other healthcare professionals in designing, implementing, and monitoring a therapeutic plan that will result in specific therapeutic outcomes with the goal of providing direct benefits for the patient. At the center of this balancing of treatment and outcomes is the patient, where medication adherence is the fulcrum that ultimately dictates success and literacy is the tool that tips the balance towards successful health outcomes.

### The Role of Literacy in Health: A Focus on Chronic Patients

Poor literacy and health literacy are serious problems with worldwide health burden and are major contributors to negative patient outcomes. In the United States, it is estimated that 47.0% of adults have difficulty understanding health-related information and lack the necessary skills to manage their health adequately (Institute of Medicine US Committee on Health Literacy, 2004); in Canada, it is an estimated 60.0% people (Canadian Council on Learning, 2008); and a recent survey of European countries suggests that 47.6% of all adults have limited health literacy skills with major differences among countries, ranging from 28.7% in the Netherlands to 62.1% in Bulgaria (Sørensen et al., 2015). For Africa, data on health literacy is scarce but it has been noted that the average literacy rate was 63% in 2015, where approximately one third of the population could not read and write (UNESCO Institute for Statistics, 2013). Clearly there is a global health literacy crisis that affects both developing and developed nations. It is also clear that improving the patient’s ability to understand and make appropriate decisions about medication-taking behavior depends on improving both literacy and health literacy by ensuring the information is both accessible and understood.

Low health literacy has also been associated with poor medication and treatment adherence in healthy and in chronic patients (Kalichman et al., 2000; Youmans and Schillinger, 2003; Ngoh, 2009).

Patients with chronic diseases and poor health literacy are generally at risk for poor health outcomes and low quality of life, and they generate a greater burden for healthcare systems. Specifically, poor health literacy is associated with higher all-cause mortality rates among patients with heart failure (Peterson et al., 2011). Furthermore, in the setting of chronic diseases such as hypertension, poor adherence limits the effectiveness of therapies proven to improve cardiovascular outcomes (Roccella, 1997) and can result in hospitalization and higher health care costs (Sokol et al., 2005). In patients living with diabetes, low health literacy has been associated with poor glycaemic control and therefore exposure to diabetes complications (retinopathy) (Bailey et al., 2014). In patients living with chronic-obstructive pulmonary disease a positive association exists with disease severity and utility of emergency healthcare services, whereas a negative association emerges with disease severity and quality of life specific to respiratory disorders, as well as helplessness in the face of illness (Omachi et al., 2013). It should be noted that some of the data on lower health literacy and nonadherence are clear (*e.g.* medication dosing), however the relationship between literacy and adherence is not equivocal as there are studies that have failed to find any relationships (Davis et al., 2006; Pignone and DeWalt, 2006).

### Chronic Patients, Health Literacy, and Medication Adherence

In 2005, 35 million people were estimated to have died from chronic diseases worldwide, representing more than 60% of all deaths globally (World Health Organization, 2005). Nearly half of all adults and approximately 8% of children (aged 5–17 years) worldwide have a chronic disease, where chronic diseases are defined broadly as conditions that last 1 year or more and require ongoing medical attention or limit activities of daily living or both (Centres for Disease Control and Prevention). Furthermore, four in ten adults have two or more chronic diseases, leaving millions requiring multiple lifelong medications for control (Buttorff et al., 2017). Poor medication adherence is a key hindrance in combating the challenges of public health related to the management of chronic conditions in both developed and developing countries (Ingersoll and Cohen, 2008). The World Health Organization defines adherence as “the extent to which a person’s behavior - taking medication, following a diet, and/or executing lifestyle changes, corresponds with agreed recommendations from a health care provider” (World Health Organization, 2003). Medication adherence is defined as the degree to which a patient adheres to the prescribed dose and interval of their medication regimen and it includes the initiation of the treatment, the implementation of the prescribed regime, and the discontinuation of the pharmacotherapy (Vrijens et al., 2012). Nonadherence to medication ranges between 25 and 50%, with higher numbers of nonadherence found in patients living in less developed countries and in patients living with chronic disease (DiMatteo, 2004). The impact of nonadherence on the patient’s health can be severe due to the reduced benefit of their medication, resulting in further disease progression or delayed recovery. This can lead to an increased risk of hospitalization and possibly death. Notably, the risk of hospitalization can be increased by over 100% in patients suffering from certain chronic conditions such as hypertension, diabetes, congestive heart failure and hypercholesterolaemia (Sullivan et al., 1990).

Indeed, in reports of medication adherence in chronically ill patients, it has consistently been reported that approximately 50% of these patients do not take their medications as prescribed (Dunbar-Jacob and Mortimer-Stephens, 2001) and in certain diseases such as hypertension, where the patient may present as asymptomatic, the incidence of non-adherence may approach 80% (Brown et al., 2016). Such findings are not shocking when considering the complexity of taking multiple medications, where patients are required to read their medication labels and associated medical information, comprehend instructions, perform numeric tasks (e.g., calculating the number of tablets to take in a day and in a single dose), and must decide what actions are required in the case of a missed dose or to monitor themselves for side effects (Youmans and Schillinger, 2003). Taken together, in order to ensure overall adherence and patient safety, a proactive approach of the health care provider is integral in identifying and preventing potential drug-related problems and resolving actual ones (Hepler and Strand, 1990), as well as communicating this complex information in the context of each patient’s own health literacy level.

### Review Aims: Medication Adherence in Chronic Patients and the Role of Pictograms

The problem of low health literacy should be considered in the broad spectrum of the current challenges of healthcare systems. Both physicians and pharmacists tend to have less time to spend on education for the patient (Gazmararian et al., 2003) so the modern healthcare systems must focus on the inter-relationship between self-management or self-efficacy from the patient, and an open dialogue with the health care team. People with low-literacy indicate that they experience a high cognitive load when required to read written drug information, which is reflected in their comments about the time and effort it takes to read and process the information (Mayer, 2002); indeed, similar findings exist in older patients living with chronic conditions (Park et al., 1994). Taken together, the aim of this review is to provide a balanced and comprehensive review outlining a number of different medication counselling and education approaches that have been used to try to improve medication adherence and health outcomes, with a specific focus on the use of clear and concise graphic illustrations—called pictograms, to help improve patient outcomes. By highlighting the current landscape of the general use and efficacy of pharmaceutical pictograms to aid in the knowledge and recall of drug-related information, as well as outlining specific medication adherence outcomes with pharmaceutical pictograms in chronic patients, the objective of this review is to describe the need for health-care providers from low- and middle-income countries to high-income countries to move beyond the traditional didactic methods of oral and verbal communication with patients in order to improve medication-taking behavior.

## Methodology

The methodology was as follows: the search was conducted with the use of Medline, EmBase, Cochrane Library and Google Scholar. We used key words like: pictogram, graphic, picture, pictorial aid, label. The search was conducted from January to March 2020. No limitation regarding the date of publication was set. No documentation regarding the collection of publications was made.

In the first step of the review, the titles of the articles were carefully checked. If the title obviously indicated that the article was not suitable for the scope of our review, the reference was rejected. If the title suggested the presence of data consistent with our interests, the publication was further analyzed. In the next step, abstract was assessed, and if still applicable, full text was analyzed. We wanted to include original studies assessing the utility, efficacy and patient feedback regarding pictograms in the context of chronic diseases among teenagers and adults, and so the criteria were defined. In our review, we aimed to present the broad spectrum of the concepts of pharmaceutical care and how pictograms can be implemented into this philosophy.

## Medication Adherence: The Current Landscape

The topic of medication adherence has been widely studied as evidenced by the fact that there are over 200 systematic reviews on the topic. According to the WHO, there are several reasons that have been identified for non-adherence, including: low health literacy, cost to patients, fear of adverse drug effects, lack of social support, etc*.*). Regarding the study design of the interventions that have assessed adherence, a Cochrane systematic review by Nieuwlaat et al. identified RCTs at lowest risk of bias, where they found that studies assessing adherence “…generally involved complex interventions with multiple components, trying to overcome barriers to adherence by means of tailored ongoing support from allied health professionals such as pharmacists, who often delivered intense education, counseling (including motivational interviewing or cognitive behavioral therapy by professionals) or daily treatment support (or both), and sometimes additional support from family or peers (Nieuwlaat et al., 2014). However, as noted but the authors, these interventions did not lead to large improvements in adherence or clinical outcomes.

Perhaps most important in any study of adherence is to first properly operationalize how this outcome is measured. In a recent review by Anderson et al., the authors identified 25 high quality systematic reviews (with a mean of 36 primary studies) assessing interventions to improve medication adherence (Anderson et al., 2020). Overall it was found that 96% of these reviews did not restrict the method for measuring medication adherence, where the main outcomes of adherence were the following: 84% of reviews had at least one primary study that employed electronic monitoring to measure adherence (e.g., mobile text messaging, pill bottles with alarm features, etc.), 84% of reviews had at least one primary study that used fill count or had at least one primary study that used patient self-report, whereas, 68% had at least one primary study that used pharmacy refill data, and 8% had at least one primary study that used patient blood levels. Overall, it was determined that the top three measures that led to improvements in adherence were: dose simplification (5 systematic reviews), electronic reminders (4 systematic reviews), and patient education (4 systematic review).

Taken together, and with the understanding that medication adherence is best promoted with the aid of health professionals such as pharmacists, findings highlighted in this section demonstrate that interventions that focus on dose simplification—with tailored ongoing support—are the most successful in improving patient outcomes for adherence (Wilhelmsen and Eriksson, 2019). For these reasons, and for the fact that pictograms have been used for many years to improve patient information concerning their medications, the following sections will discuss the potential role of pictograms in improving medication adherence, with a focus on chronic patients.

## What Is a Pictogram?

**FIGURE 1:**
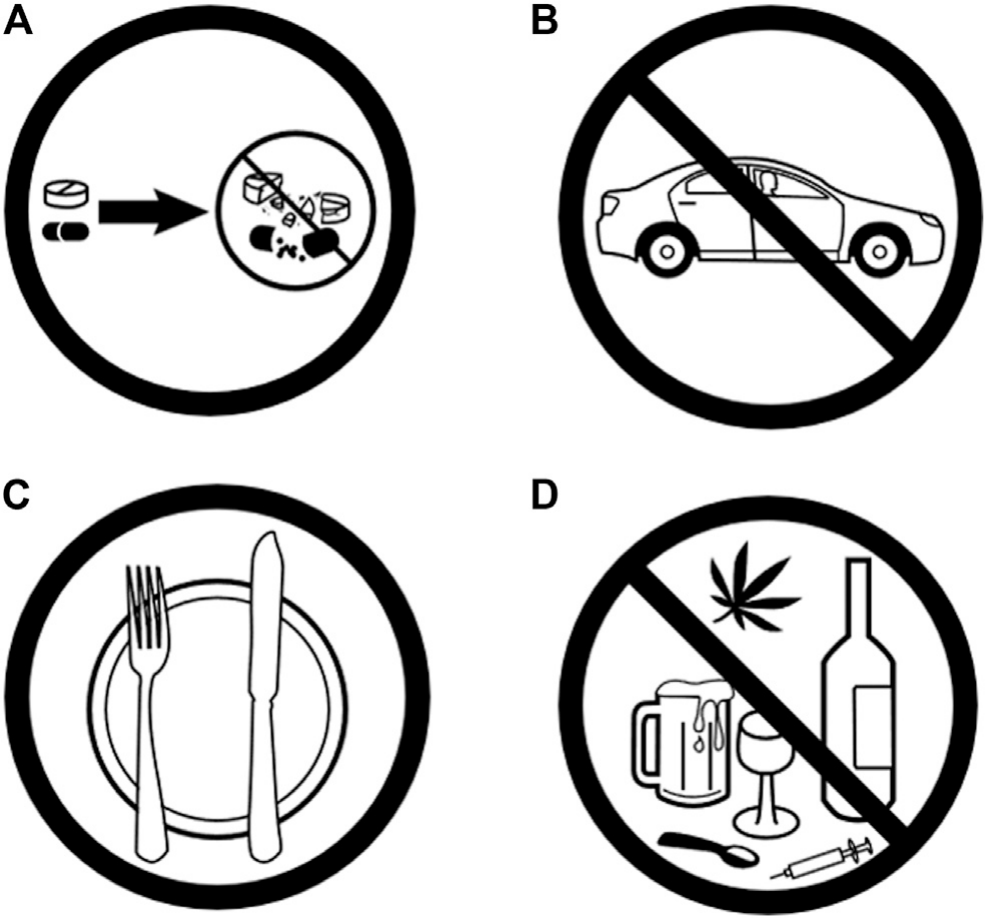
Sample examples of pictograms used in pharmaceutical care: **(A)** Do not crush; **(B)** Do not drive; **(C)** Take with food; **(D)** Do not take with alcohol or drugs. Source: https://www.fipfoundation.org/.

In general, pictograms are symbols or drawings representing a concept or idea (Peregrin, 2010). A pictogram should be considered as a two-part construct: a graphical symbol and its intended meaning. The “theory of semiotics” by Saussure introduces the distinction between the signifier (physical description of the object) and signified (its mental concept created by cultural convention) (Yang and Hsu, 2015), and importantly, the signified can have different meanings based on different cultures (Cloutier et al., 2014; Kheir et al., 2014). According to Dowse and Ehlers, “Well-designed pictograms should be simple and clear and able to convey their intended meaning to all patients, including those who are illiterate, elderly, or visually impaired.” (Dowse et al., 1998). The guiding theory of pictogram use in any field of study is that when exposed to an image, the verbal memory may be triggered by reinforcing memory traces and subsequent recall; in order to do so, the message needs to be clear, appropriate for the intended audience, and must focus on actions rather than information (see Figure 1 for examples of validated pictograms for medication counselling). Furthermore, it is important to note that pictograms should be used in combination with traditional counselling/education. Specifically, prior patient counseling by pharmacists on the intended meaning and use of pictograms has been shown to improve the pictogram effectiveness (Ngoh, 2009), a fact that has been reiterated by (Montagne, 2013) in their description of best practices for the development and assessment of pictograms (Montagne, 2013).

### Pictograms in Health-Related Research: A Focus on Chronic Patients

Why should healthcare providers consider using pictograms to convey health-related information during patient counselling? It has been reported that more than two-thirds of physicians provide written patient education materials for chronic patients (Carrier and Reschovsky, 2009), and between 40 and 80% of verbal information communicated during a health care consultation can be forgotten almost immediately (Kessels, 2003). *Introduction* of this review has already painted a picture of an epidemic of poor literacy, and despite abysmal worldwide levels of health literacy, the reading level of most health literature is above eighth grade (Cotugna et al., 2005), signifying a disparity between knowledge transfer. There is a sufficient body of evidence to show that pictograms not only make complicated patient information more attractive (Houts et al., 2006), but these images can also improve comprehension and recall of proper medication–taking behavior (Chan et al., 2015; Sletvold et al., 2020). Although the data on literacy and pictogram effectiveness is scant, results remain equivocal, where some groups have shown no impact where caregivers had adequate levels of literacy (Yin et al., 2011), whereas others have shown low and high literacy caregivers can both benefit from pictogram-based counselling (Hu et al., 2013; Tork, 2013).

There is a paucity of information on the use of pictograms in chronic patients to aid in medication adherence; however, one can see in Table 1 that the majority of studies in chronic patients are in patients living with HIV/AIDS. Although each study showed a positive outcome for the effectiveness of pictograms to improve recall and comprehension of dosing instructions, it was noted in a systematic review by Chan et al. that these are all studies with high risk of bias (Chan et al., 2015).

**TABLE 1:** Pictograms in the pharmaceutical care of chronically ill patients.

Study	N	Study setting and population characteristic	Methods and interventions	Result
**Asthma**				
Almomani et al. (2018)	219	• Jordan	• Randomised, controlled trial	• A statistically significant difference between the two groups regarding improvement in inhaler techniques after 3 months was observed for 2/4 devices: Metered dose inhaler (MDI) (*p* < 0.001) and turbohaler (*p* = 0.005)
		• Patients from an outpatient hospital pharmacy, with a diagnosis of bronchial asthma by a respiratory specialist, who used an inhaler device regularly for at least 3 months	• Intervention: Verbal consultation on the proper use of the inhalers plus pictogram medals attached to the devices	• Patients from the intervention group who used MDI and turbohaler were 7 and 5 times more likely to have improved their inhaler techniques as compared to the control group, respectively
		• ≥18 years of age	• Control: Verbal consultation only	• All intervention patients were satisfied with pictogram medals
			• Assessment at baseline and after 3 months, evaluating the inhaler techniques with a standard checklist	• No significant differences in other asthma related clinical outcomes such as adherence to medication, asthma control, or unscheduled medical intervention was observed between the two groups at study end
Wrench et al. (2019)	55	• South Africa	• Pre-post intervention study	• A statistically significant increase in the mean number of correct steps was observed: 4.6 ± 2.2 at baseline and 7.9 ± 2.7 at follow-up (*p* < 0.05)
		• Patients from a rural primary healthcare clinic, dependent on the public healthcare sector, with a diagnosis of asthma, prescribed an MDI for at least 1 month, speaking either English or isiXhosa	• Intervention: a Structured assessment of inhaler technique with the use of a 12-step checklist and a demonstration and patient education of the correct inhaler technique supported and facilitated by the illustrated study leaflet on MDI use (English or isiXhosa version)	• Statistically significant improvement of correct technique was observed in 10/12 steps controlled in the checklist
		• ≥18 years of age	• The intervention process was repeated 4 weeks later	• All except one patient enjoyed the study pictograms
**Diabetes**				
Doucette et al. (2014)	17	• Canada	• Pre-post intervention study	• High interpretation of each of the 8 “heart disease and stroke” pictograms (85% or higher) for initial and follow-up interviews was noted
		• Patients from of primary care physicians’ offices under the auspices of the Regional Health Authority’s Diabetes Education Centre diagnosed with type 2 diabetes mellitus	• Intervention: Patients were shown pictograms relating to diabetes complications, prevention, and treatment: “heart disease and stroke” (n = 8) and pictograms relating to “nerve damage’ (n = 7). Assessment of the pictogram meaning was made with a structured interview tool. Correct answers were recorded. Incorrect answers lead to patient education	• Correct interpretation of 28.6% (2/7) of “nerve damage” pictograms during the initial interview and 100% during the follow-up interview
		• 18–85 years of age	• A follow-up assessment of the recall of pictograms was planned within 8 weeks. The number of correct responses was recorded	• Overall, correct pictogram interpretation was significantly higher at the second interview (94.9 vs 82.8%; 12.2% difference; 95% confidence interval [CI], 4.7–19.7; *p* < 0.003)
				• A significant improvement in interpretation of the individual pictograms at the second interview was noted for two of the pictograms “pain medication” and “slow digestion”.
				• Among 13 participants who assessed their satisfaction with the pictograms, 11 (84.6%) were satisfied and 2 (15.4%) were neutral
Chan and Hassali, (2014)	110	• Malaysia	• Randomised, controlled trial	• All 3 groups demonstrated within-group increase of total adherence score after 4 weeks
		• Patients from outpatient pharmacy in a major general hospital with refill prescriptions of selected oral antihypertensive or antidiabetic drugs	• Intervention 1: font-enlarged labels	• Total comprehension score of pictogram-incorporated label group was significantly higher after 4 weeks (mean change 0.37, *p* = 0.010)
			• Intervention 2: pictogram-enriched labels	• Significantly higher scores for a few items in both adherence and comprehension measurements after 4 weeks were observed in the two intervention groups
			• Control: Standard labels, or pictogram-incorporated labels	• F tests indicated that all 3 groups did not significantly differ in the changes of both total adherence and comprehension scores (*p* = 0.573 and 0.069, respectively) when subjects’ age was adjusted
			• Assessment of baseline adherence, comprehension, and preferences was conducted upon recruitment; follow-up telephone interviews—after 4 weeks	• Pictogram-incorporated labels over font-enlarged labels were preferred by the elderly and those with a higher number of morbidities
Mohan et al. (2014)	200	• United States	• Randomized, controlled trial	• Patients in the PictureRx group had an overall better understanding of their medications (MUQ difference 9.9; 95% CI 5.7–14.2; *p* < 0.01)
		• Patients from a safety net clinic in Nashville, TN in a predominantly Spanish-speaking area. Patients were eligible if they were Latino, at least 18 years old, had a diagnosis of diabetes, and were prescribed at least 1 chronic medication	• Intervention: A PictureRx illustrated medication list depicted the medication, indication, and dosing instructions accompanied by plain-language bilingual text	• Each point increase in BHLS was associated with an increase of 1.1 in MUQ overall score (95% CI 0.3–2.0)
		• Patients were excluded if they could not locate their medications or had a visual acuity >20/50, had hearing deficit, dementia, psychosis, disorientation, unable to communicate in English or Spanish, lacked a regular phone number, or belonged to a special population (e.g. pregnant or prisoner)	• Control: Usual care, where patients received a written list of their medications in their preferred language, with indication but no images	• Self-reported adherence was 0.5 points higher in ARMS score n the intervention group, though not statistically significant (95% CI, -0.1–1.1)
		• 59% of patients had low health literacy tested by BHLS (Brief Health Literacy Screen)	• Outcomes were assessed by telephone 1 week later. The Medication Understanding Questionnaire was used to assess the patient’s ability to report indication, strength, dosing and frequency for their medication regimen	• Patients who received the PictureRx intervention reported very high satisfaction. 99% reported that the tool was easy clear, easy to read, and helped them remember which medicines to take (96.9%) and when to take them (96.9%)
		• 71% of patients had not graduated high school	• Secondary outcomes assessed were self-reported adherence and satisfaction. Medication adherence was assessed by the Adherence to Refills and Medications Scale (ARMS)	
Negarandeh et al. (2013)	127	• Iran	• Randomized, controlled trial	• Mean scores of knowledge, adherence to medication and adherence to dietary regimen were significantly higher in both intervention groups compared to control (*p* < 0.05)
		• Patients recruited from a secondary level diabetes clinic in Saqqez, Kurdistan with a diagnosis of type 2 diabetes for more than 6 months and had low health literacy (defined by a score of 59 or lower on TOFHLA), were at least 18 years old, and had no visual, mental, or learning disabilities	• Intervention 1: Education based on a teach-back strategy during three weekly sessions lasting 20 min each. The patient also received written instructions of important information	• There were no significant differences in knowledge or adherence between the two intervention groups
		• Patients were excluded if they had participated in previous diabetes education research	• Intervention 2: Patients received education with illustrated content during three weekly sessions lasting 20 min each	
			• Control: Patients received usual care which involved diabetes education presented in a brochure. Questions were answered by a community health nurse, similarly to the intervention groups	
**AIDS**				
Mansoor and Dowse, (2006)	120	• South Africa	• Randomised, controlled trial	• In the group receiving materials incorporating simple text and pictograms a significant increase in adherence to therapy was noted, whereas a non-significant increase in adherence was observed in the group receiving more complex information (measured both by the self-report and the tablet count)
		• Patients from local primary healthcare out-patient clinics, HIV-positive patients on chronic cotrimoxazole therapy, from a variety of educational backgrounds, able to read and understand either English or isiXhosa	• Two different PILs were designed for co-trimoxazole tablets and were available in both English and isiXhosa	• Combined results from the self-report and tablet count found that the overall mean percentage adherence of the participants receiving Intervention 2 (88.3%) was significantly higher than those receiving Intervention 1 (73.6%), and the control group (67.7%) (*p* < 0.05)
		• > 16 years of age	• Intervention 1: Longer text-only PIL	• Significantly more participants receiving Intervention 2 (92.5%) obtained 100% adherence when compared with the control group (70.0%) (*p* < 0.05)
			• Intervention 2: Simple PIL with pictograms	• The results from the tablet count showed that the mean percentage adherence was significantly higher in participants who received Intervention 2 (86.5%) when compared with the controls (65.1%) or Intervention 1 (70.1%) (*p* < 0.05)
			• Control: No PIL	
			• Adherence to therapy was assessed using two methods: Self-report and tablet count ∼14 days later	
Mansoor and Dowse, (2007)	120	• South Africa	• Randomised, controlled trial	• The mean percentage for knowledge of medicines was significantly higher in the group that received the simple PIL incorporating pictograms (76.3%), compared with both the control group (43.3%) and the group who received the longer, text-only PIL (50.9%) (*p* < 0.05)
		• Patients from local primary healthcare out-patient clinics, HIV-positive patients on chronic cotrimoxazole therapy, able to read and understand either English or isiXhosa	• Intervention 1: Text-only PIL, longer and more complex	
		• > 16 years of age	• Intervention 2: a simple, shorter PIL that incorporated pictograms and text	
			• Control: No PIL	
			• Medicines knowledge was investigated in an interview ∼14 days later	
Wilby et al. (2011)	82	• Canada	• Randomised, controlled trial	• Majority of the targeted pieces of information (88%) in the intervention group were correctly identified at follow-up, while only 2% in the controls (*p* < 0.0001)
		• Patients from an ambulatory pharmacy, HIV-positive patients who are receiving a new prescription for an antiretroviral medication	• Intervention: pictogram-enhanced information	• Majority of the intervention patients (79%) recalled properly all targeted information versus none of patients in the control group (*p* < 0.0001)
		• ≥19 years of age	• Control: Standard counselling	• The results were dependent of the fact that each pictogram was explained to patients prior to use
			• Evaluation of recall at first follow-up visit	
			• Evaluation of the recall at the next follow-up appointment at the ambulatory pharmacy	
			• Correct/incorrect response were recorded	
Kalichman et al. (2013)	446	• United States	• Randomized clinical trial	• Participants with marginal health literacy receiving Intervention 1 and Intervention 2 demonstrated greater adherence and undetectable HIV viral loads compared to controls
		• Patients from AIDS services and community outreach, with HIV and receiving antiretroviral therapy, with marginal and lower health literacy levels	• Intervention 1: Pictograph-guided adherence counselling	• Participants with lower health literacy skills in the control group demonstrated greater adherence compared to the two adherence counselling groups
			• Intervention 2: Standard adherence counselling	
			• Control: general health improvement counselling	
			• Unannounced assessment: pill count adherence and blood plasma viral load 9-months post-intervention	
Dowse et al. (2014)	116	• South Africa	• Randomised, controlled study	• Intervention patients presented a significant knowledge increase over the 6-month period (62.0–94.4%), and an improvement at each subsequent interview was noted. No improvement was observed in the control group
		• Patients from rural clinics, HIV/AIDS patients who had been taking a first-line ARV regimen for less than 3 months, had isiXhosa as their home language, and had a maximum of 10 years of schooling	• Intervention: Standard care plus simple pre-tested PIL containing both text and illustrations	• Side effect knowledge, which was the lowest (50–56%) at baseline increased in the intervention group to 92%
		• ≥18 years of age	• Control: Standard care	• Other medicine-related knowledge at baseline (57–67%) improved significantly (93%), which was sustained over 6 months
			• HIV and medicines-related knowledge was evaluated at baseline, one, three, and 6 months post-intervention; self-efficacy was assessed over 6 months	• A large intervention effect was observed (Cohen’s d values post-baseline were 1.36–2.18)
				• A significant improvement over 6 months in self-efficacy was observed in the intervention group but not in controls
				• At baseline, patients with ≤3 years of education had lower knowledge and self-efficacy. However, this was not reported post-intervention, which was attributed by the authors to the PIL mitigating the limited education effect
				• In the intervention group knowledge and self-efficacy were significantly correlated
Monroe et al. (2018)	46	• United States	• Randomized controlled study	• There was a trend towards higher adherence to medications for HIV as compared with hypertension/diabetes medications (*p* = 0.07)
		• Patients from the johns Hopkins HIV clinic, patients with HIV and diabetes and/or hypertension attending a clinic for underserved patients and those at risk for poor health outcomes	• Intervention: Pictorial aid: a photographic representation of the medications, the indications, and the dosing schedule	• The intervention was feasible to implement and satisfaction with the intervention was high
		• Patients using the HIV clinic pharmacy for all prescriptions, being prescribed medications for HIV and diabetes and/or hypertension for ≥6 months, and being prescribed ≥5 different medications per day total (for any condition); speaking English	• Control: Standard clinic visit discharge medication list	
		• ≥ 18 years of age	• Assessment of the adherence to antiretroviral therapy (ART) for HIV and therapy for diabetes or hypertension was compared	
Browne et al. (2019)	116	• South Africa	• Randomised, controlled study	• The mean side effect knowledge increased from 45.9% (baseline) to 95.7% (after 3 months) in the intervention group (*p* < 0.0001)
		• Patients from local public sector clinics, HIV patients taking antiretroviral drugs, patients with limited literacy, isiXhosa-speaking	• Intervention: Standard care plus illustrated information: Side-effect pictograms, combined with simple text, incorporated into a side effects panel within an ARV information leaflet	• Knowledge did not change significantly in the control group
			• Control: Standard care	• Pictogram interpretation was good
			• Side-effect knowledge was assessed at baseline. Interpretation of side-effect pictograms was evaluated after 1 month. Knowledge was re-tested after one and 3 months	• All patients found the pictograms clear and useful, and endorsed their routine use
**Chronic kidney Disease**				
Mateti et al. (2015)	81	• India	• Quasi-experimental pre- and post-test	• The overall user testing knowledge assessment mean scores significantly improved from 44.25 to 69.62 (*p* < 0.001)
		• Patients from haemodialysis (HD) units of academic, government, and corporate hospitals, HD patients on pharmaceutical care group with minimum primary educational background, patients undergoing HD continuously for 3 months	• Without control group	
		• 18–75 years of age	• Usability testing of the pictogram-based PILs	
Cardiac disorders				
Zerafa et al. (2011)	80	• Malta	• Randomised, controlled trial (although called a case-controlled study in the manuscript)	• Patients in the intervention group had a higher mean percentage compliance score (88%) than the controls (66%) (*p* < 0.05)
		• Patients who underwent coronary artery bypass or heart valve surgery at the cardiac Surgical Ward and Medical outpatients clinic of Mater Dei Hospital, Birkirkara, Malta, able to communicate with the investigator; mentally competent	• Intervention: a chart with pictorial explanation of the time of day together with a colourful photograph of each tablet prescribed and counselled to comply to oral analgesia and exercise and also on the avoidance of alcohol and smoking during the recovery period	
		• >18 years of age	• Control: Usual care without the pharmacist intervention	
			• All patients were re-interviewed 8 weeks after discharge	
Kripalani et al. (2012)	435	• United States	• Randomised, controlled trial	• Among those that CMG could be calculated 138 (32.9%) had CMG<0.20 during follow-up and were considered adherent
		• Patients from an inner-city primary care clinic, adults with coronary heart disease	• 2 × 2 factorial design: Usual care, refill, reminder postcards, illustrated medication schedules, or both interventions for 1 year	• Overall, adherence rate did not differ significantly across treatments: 31.2% in usual care, 28.3% with mailed refill reminders, 34.2% with illustrated medication schedules, and 36.9% with both interventions
			• Cardiovascular medication refill adherence was assessed by the cumulative medication gap (CMG)	• In post-hoc analyses, illustrated medication schedules were found to led to significantly greater odds of adherence among patients who at baseline had >8 medications (OR = 2.2; 95% CI, 1.21–4.04) or low self-efficacy for managing medications (OR = 2.15; 95% CI, 1.11–4.16); a trend was found among patients who reported non-adherence at baseline (OR = 1.89; 95% CI, 0.99–3.60)
Hawkins and Firek, (2014)	27	• United States	• Crossover study	• Patients medication adherence significantly improved from pre-intervention to post-intervention (t (26) = 2.16, *p* < 0.05)
		• Patients from a large Veterans’ Administration facility in southern California who presented to the VA outpatient heart failure clinic, with congestive heart failure (CHF) and cognitive impairment	• Pre-intervention: Subjects brought their prescribed medications for a pill count, and returned after 30 days for a repeat pill count to assess baseline adherence	• Patient acceptance of the intervention was high, with 74.1% of participants indicating the medication sheet was very helpful in remembering to take medications and filling medication boxes
		• Participants 18 years old or older, had a diagnosis of CHF, and screened positive for cognitive impairment using the Saint Louis University Mental Status (SLUMS) exam	• Post-intervention: customized pictorial medication sheet with brief instructions on use, and optional CADEX Pocket Pill Box with vibrating alarms. Pictorial medication sheets included images on the participant’s current medications printed in full colour and dose range, arranged in columns for morning, noon, and evening. A brief description of the medication name, indication, and dose were included. Pill count was assessed after 30 and 60 days	
		• Patients excluded if they had severe functional limitations, acutely decompensated CHF, dementia requiring a caregiver, severe mental illness such as schizophrenia, or a life expectancy of less than 6 months		
Machtinger et al. (2007)	147	• United States	• Randomized, controlled trial	• Intervention subjects achieved anticoagulation control more rapidly than control subjects (median 28 vs 42 days; hazard ratio [HR] 1.43; CI 1.00, 2.06)
		• Patients from a pharmacist-staffed anticoagulation clinic at San Francisco General Hospital (SFGH) receiving chronic warfarin	• Intervention: Visual medication schedule at each visit with digitized images of the patient’s warfarin regimen printed on a weeklong calendar, plus standard care	• The intervention showed significant benefit in subjects with baseline regimen discordance (median 28 vs 49 days; HR 1.92; CI 1.08, 3.39) but the benefit was not significant among subjects with baseline concordance (median 28 vs 35 days; HR 1.14; CI 0.71–1.83)
		• At least 18 years old, spoke English, Spanish or Cantonese, were taking warfarin for at least 3 months, had an INR outside the therapeutic range (either 2.0–3.0 or 2.5–3.5) within 5 days prior to enrolment	• Control: Standard care, which included standard medication counselling and follow-up in the anticoagulation clinic and consultation and medication management by pharmacists with expertise in anticoagulation	
		• Patients excluded if they had a psychiatric disorder, dementia, blindness, aphasia, were too ill to participate, could not communicate independently, had corrected vision of 20/100 or worse, used pill boxes filled by health care professionals, managed their warfarin by telephone, expected to stop taking warfarin within 90 days, or were not using warfarin preparations on the SFGH formulary		

TOFHLA, Test of Functional Health Literacy in Adults.

As seen in Table 1, there were several underlying themes that emerged in these interventions that deployed pictograms: the impact on pharmacotherapy safety, patient awareness and improvement of treatment results, patient involvement in the therapeutic process, and patient communication and trust in the delivery of their healthcare. It is worthwhile to note that a recent systematic review by Nguyen et al. identified at least 43 validated self-report adherence scales (Nguyen et al., 2014), indicating that it is crucial to consider the tool being used to assess the outcome of adherence, as well as the quality of the pictograms being tested. Overall, there is no single measure that can assess all the behaviors involved in being adherent to medication(s) and how this can impact patient outcomes. Selecting two (or more) medication adherence measures such as can be seen in Table 1 can allow strengths of one method (indirect methods of verbally explaining medication instructions) to help compensate putative weakness and to more accurately capture the information needed to determine adherence levels when incorporating another method (such as pictograms).

In patients living with chronic illness, data suggests that individuals with low health literacy can benefit more from interventions employing pictograms (Machtinger et al., 2007; Negarandeh et al., 2013; Mohan et al., 2014; Phimarn et al., 2019), whereas, individuals with high health literacy do not seem to benefit to the same degree (Kripalani et al., 2012). Table 1 summarizes the results of studies on the use of pictograms among chronically ill patients in distinction for the following diseases: asthma (*n* = 2), diabetes (*n* = 4), AIDS (*n* = 7), chronic kidney disease (*n* = 1), and cardiac disorders (*n* = 4). Studies focusing on the elderly were analyzed separately (*n* = 3). Studies focusing on self-efficacy were analyzed separately (*n* = 2). The data obtained in the selected studies indicate that the use of pictograms significantly increased various aspects of disease self-management of the patients with chronic conditions. However, not all pictogram interventions were found to be effective, where several groups have failed to find a significant effect of pictograms in helping with medication adherence in patients living with chronic illness (Kripalani et al., 2012; Advani et al., 2013; Chan and Hassali, 2014).

In our work, apart from comprehensive review on the impact of pictograms on adherence in chronically ill patients, we also would like to point out some methodological issues, which may be an obstacle to a reliable assessment of the impact of pictograms on adherence.

Publications summarize in Table 1 describes the role of pictograms in chronically ill patients, but from methodological point of view, they are different in many respects. First of all, the results were obtained in many different countries and among subjects with different level of literacy. As we stated previously these factors can have a considerable impact on the usefulness and efficacy of pictograms. Additionally to this, interventions were carried out in patients with different chronic diseases. Moreover, the number of patients participating in assessing studies varied a lot (*n* = 17–446). Apart from that, these studies lacked information of the clarity and appropriateness of pictograms chosen. There was also a lack of explanations on the validity and reliability of instruments used. One should also pay attention to the differences in the methods of patient education. Interventions described in cited publications were carried out both in pharmacies and clinics, so we can anticipate many differences in approach to education as well as the amount of time devoted to the patient.

These all above described factors impact on heterogenicity of published trials and consequently have an impact on the possibility of comparing obtained results, drawing conclusions and preparing more credible publications like metanalysis. Future research should also devote more attention to aforementioned limitations of the included studies. Maybe good tool, which can contribute to overcome these methodological problems is developing specific protocols that will be used in further studies. That protocols could allow collect more coherent results and precise assessment of the impact of pictograms on adherence.

### Importance of Pictogram Design and Evaluation: Fundamental Concepts, Issues, and Problems

#### Best Practices in Pictogram Design

Pictograms, when properly designed and validated, have been implemented to help convey medication information in many health-related settings and humanitarian missions (e.g., hospitals, pharmacies, temporary aid stations, etc.) with varying degrees of successful outcomes (Houts et al., 2006; Sorfleet et al., 2009; Dowse et al., 2014). But in order to make any conclusions on the effectiveness of pictograms on medication adherence, and prior to having to consider how to answer a research question with a large and expensive randomized trial, one first needs to be sure the image meets specific standards of best practice in pictogram design.

Researchers in the field of health-related pictograms should consider the following. First, and most important to ensuring best practices are being followed, is the rigorous and time consuming process of designing new pictograms. Pictograms must go through a systematic design process, whereby images are piloted and tested on scales of transparency (or “guessability of meaning”) and translucency (“agreeability of intended meaning”), each of which contribute to independent elements that come together to determine the overall comprehensibility of the pictogram (Vaillancourt et al., 2017), which can also be evaluated with open choice face-to-face methods (Mansoor and Dowse, 2003). It is important to note that there are differences in patient populations to consider (e.g., education, beliefs, attitudes, etc*.*) in this initial process of testing and design (Dowse and Ehlers, 2003). In particular, many studies have now demonstrated the importance of considering differences in pictorial interpretation between countries, and several studies have also demonstrated cultural differences within countries, and these differences can determine whether the information relayed in the pictogram is correctly understood (Doak et al., 1996; Ehlers et al., 2004; Grenier et al., 2011). As such, if the researcher is to ensure an effective evaluation process for pictogram design that can provide data that is both valid and reliable, it is integral to have the target population involved at all stages of the design process.

Furthermore, in order to be able to systematically evaluate differences in study outcomes in the field of health-related pictogram research there are several other important elements to consider to ensure that best practices in pictogram design are followed (for extensive details see: Dowse, 2020; Abdullah et al., 2006; Mansoor and Dowse, 2004). These are guiding documents that should serve to help groups in health-related research to produce high quality pictograms with optimal legibility and visibility. Sletvold et al. (2020) concluded that studies assessing the value of pharmaceutical pictograms must establish best practices in the design and use of pictograms to ensure we can isolate the independent effects of pictograms on medication adherence (Sletvold et al., 2020).

### Best Practices in Pictogram Evaluation

An important consideration for the successful evaluation of pictograms in the delivery of health information is to ensure that the healthcare professionals who will ultimately deliver the education and counselling are involved in the entire process of design and evaluation. For example, when implementing pharmaceutical pictograms that help with medication adherence, patients generally still need to be taught how to interpret the pictograms (Ngoh, 2009), and these increased task demands on the pharmacist must be perceived as a beneficial use of time and resources in order to get the most benefit for the patient. Much research in the study of pictograms in the field of health information—and in particular research on medication adherence—still does not explicitly specify if pharmacists were involved in the design or implementation of the specific pictogram intervention. Results described by Wilhelmsen & Eriksson (2019) in a recent systematic review possibly speak to this importance, where they noted: “…that interventions delivered by pharmacists and nurses showed a better result in improving adherence and outcomes than interventions delivered by general practitioners (Wilhelmsen and Eriksson, 2019). Although this is not direct evidence for the case, future research can benefit from a standardization process whereby these details are systematically documented, for example, when trials are registered. Finally, once best practices have been employed in the design and evaluation of the pharmaceutical pictograms, the research team is ready to design a randomized controlled trial that can isolate the independent effects of pictograms on, for example, medication adherence. As noted by (Anderson et al., 2020) systematic overview of systematic reviews, much of the research on medication adherence is highly heterogeneous in study designs, settings, and methods (Anderson et al., 2020), so attention must be focused on the fidelity of design to ensure reproducibility of the data. These summaries of fundamental problems were echoed by (Sletvod et al., 2020), where the authors concluded that heterogeneity in the design and conduct of the current landscape of RCTs examining pictograms in medication adherence preclude any meta-analysis of observed pictogram effects (Sletvold et al., 2020).

## Conclusions and Future Perspectives

On the basis of the reviewed data, we conclude that effective spoken and written communication of information about medicine, in combination with health-related pictograms, can lead to improved treatment outcomes for the patient. Low health literacy levels negatively affect patients—including chronic patients—across the continuum of care. With this in mind, pharmacists and health care-providers are well-situated to ensure that patients adhere to their medication regimens correctly, especially so for lower health literacy populations. Chronically ill patients are a population that can benefit from interventions (e.g. “teach back” and Ask Me 3™) because their pharmacotherapy is often a life-long challenge, which often requires coping with complex daily multi-drug regimens that can require high levels of literacy and self-efficacy.

The current review highlighted adherence in chronic patients. Due to a paucity of research on pictograms and medication adherence in this population and due to the lack of best practice in much of the existing research, we could not conclude that these patients benefitted any more or less from pictogram interventions. Indeed, in the recent systematic review by Sletvold et al. (2020) the authors found a possible effect of pictograms on medication adherence, but in order to make stronger conclusions, researchers in this field need to follow best practices not only in the design and evaluation of health-related pictograms, but also in the design of strong RCTs that can isolate the independent effects of pictograms on study outcomes (Sletvold et al., 2020). Taken together, research in the field of health-related pictograms and medication adherence should focus on creating content that focuses on dose simplification and electronic reminders. By adhering to best practices in the design, testing, and evaluation of pictograms, we can strengthen future findings from this exciting and visually stimulating field of health-related research.
